# Hippocampal transcriptome reveals novel targets of FASD pathogenesis

**DOI:** 10.1002/brb3.1334

**Published:** 2019-05-29

**Authors:** Raine Lunde‐Young, Josue Ramirez, Vishal Naik, Marcus Orzabal, Jehoon Lee, Kranti Konganti, Andrew Hillhouse, David Threadgill, Jayanth Ramadoss

**Affiliations:** ^1^ Department of Veterinary Physiology and Pharmacology, College of Veterinary Medicine and Biomedical Sciences Texas A&M University College Station Texas; ^2^ Department of Veterinary Pathobiology, College of Veterinary Medicine and Biomedical Sciences Texas A&M University College Station Texas

**Keywords:** brain, hippocampus, nitric oxide, pregnancy, teratology

## Abstract

**Introduction:**

Prenatal alcohol exposure can contribute to fetal alcohol spectrum disorders (FASD), characterized by a myriad of developmental impairments affecting behavior and cognition. Studies show that many of these functional impairments are associated with the hippocampus, a structure exhibiting exquisite vulnerability to developmental alcohol exposure and critically implicated in learning and memory; however, mechanisms underlying alcohol‐induced hippocampal deficits remain poorly understood. By utilizing a high‐throughput RNA‐sequencing (RNA‐seq) approach to address the neurobiological and molecular basis of prenatal alcohol‐induced hippocampal functional deficits, we hypothesized that chronic binge prenatal alcohol exposure alters gene expression and global molecular pathways in the fetal hippocampus.

**Methods:**

Timed‐pregnant Sprague–Dawley rats were randomly assigned to a pair‐fed control (PF) or binge alcohol (ALC) treatment group on gestational day (GD) 4. ALC dams acclimatized from GDs 5–10 with a daily treatment of 4.5 g/kg alcohol and subsequently received 6 g/kg on GDs 11–20. PF dams received a once daily maltose dextrin gavage on GDs 5–20, isocalorically matching ALC counterparts. On GD 21, bilateral hippocampi were dissected, flash frozen, and stored at −80^°^C. Total RNA was then isolated from homogenized tissues. Samples were normalized to ~4nM and pooled equally. Sequencing was performed by Illumina NextSeq 500 on a 75 cycle, single‐end sequencing run.

**Results:**

RNA‐seq identified 13,388 genes, of these, 76 genes showed a significant difference (*p* < 0.05, log2 fold change ≥2) in expression between the PF and ALC groups. Forty‐nine genes showed sex‐dependent dysregulation; IPA analysis showed among female offspring, dysregulated pathways included proline and citrulline biosynthesis, whereas in males, xenobiotic metabolism signaling and alaninine biosynthesis etc. were altered.

**Conclusion:**

We conclude that chronic binge alcohol exposure during pregnancy dysregulates fetal hippocampal gene expression in a sex‐specific manner. Identification of subtle, transcriptome‐level dysregulation in hippocampal molecular pathways offers potential mechanistic insights underlying FASD pathogenesis.

## 1 INTRODUCTION

Fetal alcohol spectrum disorders (FASD) collectively describe an array of physical abnormalities, central nervous system disruptions, and cognitive and behavioral deficits induced by prenatal alcohol exposure (Riley, Infante, & Warren, 2011; Sokol, Delaney‐Black, & Nordstrom, 2003). In the United States, more than 30% of pregnancies are estimated to be affected by prenatal alcohol exposure (Ethen et al., 2009), and one in 10 pregnant women report alcohol consumption in the past 30 days (Tan, Denny, Cheal, Sniezek, & Kanny, 2015). A recent study estimates that FASD prevalence in the U.S. populations may range from 3% up to 9% (May et al., 2018). A myriad of factors influence phenotypic severity within FASD, including timing, dose, and duration of exposure, as well as maternal nutrition, genetic susceptibility of both the mother and fetus, and parental history of substance use disorder (Maier & West, 2001; May et al., 2013; May & Gossage, 2011; Smith, Garic, Berres, & Flentke, 2014). These variables, coupled with the fact that in utero alcohol exposure impairs nearly every developing organ system, attribute to the wide‐ranging variation in the presentation and severity of FASD phenotypes among affected individuals (Caputo, Wood, & Jabbour, 2016; Hofer & Burd, 2009; Popova et al., 2016). Birth defects resulting from prenatal alcohol exposure are persistent and lifelong, with profound socioeconomic consequences (Thanh, Jonsson, Dennett, & Jacobs, 2011); currently no approved pharmacologic therapy exists (Spohr & Steinhausen, 2008). Targets of prenatal alcohol exposure and its pharmacokinetics are complex in nature, and thus to date, the molecular mechanisms underlying FASD pathogenesis remain insufficiently understood (Burd, 2016).

The fetal brain is one of the most well‐studied targets of gestational alcohol exposure. Human and animal model studies have implicated the developing hippocampus, a structure associated with learning and memory function, as exquisitely vulnerable to alcohol‐induced developmental damage (Dudek, Skocic, Sheard, & Rovet, 2014; Lewis et al., 2015). In animal models, gestational alcohol‐induced alterations to hippocampal synaptic plasticity have been extensively studied (Bhattacharya et al., 2015; Fontaine, Patten, Sickmann, Helfer, & Christie, 2016), as well as alcohol‐induced alterations to hippocampal synaptic activity (Kajimoto et al., 2016) and regional and cellular morphology (Berman & Hannigan, 2000; Perez, Villanueva, & Salas, 1991; Ramos, Evrard, Tagliaferro, TricÁrico, & Brusco, 2002). In humans, prenatal alcohol exposure produces asymmetrical reduction in hippocampal volume, impaired spatial recall, delayed reproduction of a spatial figure, impaired place learning, delayed recognition, and verbal learning tasks relative to controls (Autti‐Rämö et al., 2002; Hamilton, Kodituwakku, Sutherland, & Savage, 2003; Willoughby, Sheard, Nash, & Rovet, 2008).

A limited number of FASD animal model studies have reported alterations in the hippocampal transcriptome using DNA microarray analysis (Chater‐Diehl, Laufer, Castellani, Alberry, & Singh, 2016; Lussier, Stepien, Weinberg, & Kobor, 2015; Mandal, Park, Jung, & Chai, 2015). One study reported that developmental alcohol dysregulates several genes implicated in the nervous system development (*Nova1, Ntng1, Neurog2, and Fexfs*) (Mandal et al., 2015), and another reports that alcohol alters hippocampal gene expression, DNA methylation, and histone methylation in free radical scavenging networks in offspring 70 days after birth (Chater‐Diehl et al., 2016). FASD studies have also shown altered hippocampal DNA methylation and gene expression on postnatal day (PND) 28 corresponding with asymmetrical hippocampal volume on PND 60 in offspring exposed to alcohol during early neurulation (GDs 0.5–8) (Marjonen et al., 2015), and that alcohol exposure on GDs 8–21 dysregulates several candidate genes (*Gabrb3, Ube3a, Mecp2, and SLC25a12*) that overlap with autism spectrum disorders and concurrently produces adverse hippocampal learning outcomes in adult offspring (Tunc‐Ozcan, Ullmann, Shukla, & Redei, 2013; Tunc‐Ozcan, Wert, Lim, Ferreira, & Redei, 2018).

These studies largely utilize microarrays to assess gene expression in mature offspring, a time when hippocampal‐based learning outcomes can be effectively assessed. Our study is unique as it is the first to utilize high‐throughput next‐generation (next‐gen) RNA deep‐sequencing (RNA‐seq) to examine a more thorough, dynamic range of transcriptome‐wide effects of chronic prenatal binge alcohol exposure on the developing hippocampus. Alcohol‐induced dysregulation to the hippocampal transcriptome during pregnancy could substantially impair hippocampal development, and associated adverse consequences may impair juvenile learning outcomes that could persist into adulthood. It is essential to understand the fully alcohol‐induced hippocampal transcriptome dysregulation early in life so that targeted intervention strategies may be effectively applied as soon as possible. However, microarray analysis is restricted in its ability to detect differentially expressed genes due to factors such as high background levels caused by cross‐hybridization and signal saturation, and also lacks sensitivity for genes with very high or low expression levels (Wang, Gerstein, & Snyder, 2009). By utilizing next‐gen RNA‐Seq as a strategic means for investigating multi‐mechanistic actions of alcohol on holistic gene expression of target organ structures, a much larger dynamic range of differentially expressed hippocampal genes can be detected (Wang et al., 2009). Since alcohol has been shown to affect various aspects of fetal hippocampal development, (Boschen & Klintsova, 2017; Gil‐Mohapel et al., 2011; Mantha, Kleiber, & Singh, 2013), it is imperative to further discern how gestational alcohol exposure alters this structure at the level of the transcriptome so that we may understand mechanisms underlying neuropathogenesis and develop appropriately targeted intervention strategies.

Our model of FASD has previously shown distinct dysregulation of amino acid homeostasis and the protein signature in the fetal hippocampus (Davis‐Anderson et al., 2018). The purpose of the study was to discern the alterations of fetal hippocampal gene expression and their associated pathways in response to maternal alcohol exposure. We hypothesized that chronic gestational alcohol exposure alters fetal hippocampal gene expression and their related global canonical pathways.

## 2 MATERIALS AND METHODS

### 2.1 Animals

All experimental procedures were in accordance with the National Institutes of Health guidelines (NIH Publication No. 85–23, revised 1996) with approval by the Animal Care and Use Committee at Texas A&M University. Timed‐pregnant Sprague–Dawley rats were purchased from Charles River (Wilmington, MA), and were housed in a temperature‐controlled room (23°C) with a 12:12‐hr light–dark cycle. Rats were assigned to a pair‐fed control (PF) group (*n* = 6 dams) or an alcohol (ALC) treatment group (*n* = 6 dams) on GD 4. The ALC‐treated animals acclimatized via a once daily orogastric gavage of a 4.5 g/kg (22.5% wt/v, peak BAC, 216 mg/dl) alcohol dose from GDs 5–10, and progressed to a 6 g/kg dose (28.5% wt/v, peak BAC, 289 mg/dl) (Davis‐Anderson et al., 2018) from GDs 11–20. The PF animals were isocalorically matched to the ALCs by daily dosing with a gavage of maltose dextrin to account for calories derived from alcohol. The exposure regimen utilized in this study is based on both reported binge alcohol consumption patterns in pregnant women and binge exposure patterns implemented across FASD animal models (Caetano, Ramisetty‐Mikler, Floyd, & McGrath, 2006; Church & Gerkin, 1988; Cudd, Chen, & West, 2002; May et al., 2013; Ryan, Williams, & Thomas, 2008; Thomas, Idrus, Monk, & Dominguez, 2010). All rats were weighed prior to the start of the study, and each treatment animal was yoked with a control animal of similar weight throughout the duration of the study. Feed intake in both groups was measured daily and the amount of diet consumed by the ALC animals was matched to the diet administered to PF animals. There was no significant maternal weight difference between treatment groups. Animals were sacrificed on GD 21, one day after the last alcohol exposure.

### 2.2 Fetal hippocampal isolation

Fetal brain tissue was collected from an equal number of male and female offspring within each treatment group. Brains were extracted under a dissection microscope via craniotomy and were serially washed in cold phosphate buffered saline (PBS), meninges were removed, and bilateral hippocampi were microdissected in ice‐cold HEPES buffer. Individual samples were then flash frozen and stored at −80 ^◦^C until analyses. One pair of male or female hippocampi from each dam was utilized for analysis.

### 2.3 Sample preparation

Each tissue sample was homogenized in TRIzol® Reagent and total RNA was isolated according to manufacturer's protocol (Invitrogen; Carlsbad, CA). Prior to analysis, RNA quality was assessed using an Agilent TapeStation RNA assay. Whole‐genome RNA transcripts were quantified via Qubit Fluorometric assay and subsequently all samples were normalized to an equivalent starting concentration. Sequencing libraries were prepared using the TruSeq Stranded mRNA Library Prep kit (Illumina; San Diego, CA). Each sample was uniquely indexed (barcoded) to allow for pooling of all samples in a single sequencing run. Library size and quality were then assessed with an Agilent TapeStation D1000 DNA assay. Samples were normalized to ~4nM and pooled equally. Sequencing was performed on an Illumina NextSeq 500 running with a 75 cycle, single‐end sequencing run.

### 2.4 Bioinformatics

Raw RNA‐sequence data were analyzed to identify significant differences in gene expression between the PF and ALC treatment groups, sex‐dependent expression differences between these treatment groups, and the global biological pathways associated with disruption of these hippocampal genes. A total of approximately 142 million reads were evaluated and trimmed of all adapter sequences and low quality bases using Trimmomatic read trimmer (Bolger, Lohse, & Usadel, 2014). Using Trimmomatic and the corresponding adapter sequences file for Illumina, reads were scanned with a sliding window of 5, cutting when the average quality per base drops below 20, then trimming reads at the beginning and end if base quality drops below 20, and finally dropping reads if the read length is less than 50. This resulted in 131 million filtered reads (approximately 92%), of which a total of 128 million filtered reads (approximately 97%) were mapped to the *Rattus norvegicus* (rn5) genome assembly. Read mapping for our samples was performed using HISAT genomic analysis software platform version 2.0.5 (Kim, Langmead, & Salzberg, 2015). Transcript‐wise counts were generated using the featureCounts tool from the SUBREAD high‐performance read alignment package (Liao, Smyth, & Shi, 2013). Differential gene expression tests were then performed using DESeq2 software following the guidelines recommended by Love and colleagues (Love, Huber, & Anders, 2014). Heat map and volcano plots were generated from this processed data using the R programming language. The resulting gene expression values for genes that met statistical significance criteria were uploaded to INGENUITY^®^ Pathways (QIAGEN, Venlo, Netherlands; Application Build 261899, Content Version 18030641) for biological pathway analysis. A core analysis was used to identify top canonical pathways effected by the alcohol treatment. Filters utilized for this analysis include species, confidence, mutation, and molecule type.

### 2.5 Statistical analyses

Raw read counts for each gene in each hippocampal sample were utilized as input into DESeq2, which modeled the read counts as following a negative binomial distribution, with a mean representing the read concentration per gene. This mean was scaled by a normalization factor (median‐of‐ratios) to account for differences in sequencing depth between samples. During independent filtering, DESeq2 used the average expression strength of each gene, across all samples as its filter criteria, and omitted all genes with mean normalized counts below a filtering threshold from multiple testing adjustments. The geneset that satisfied −2 > log2(fold change) >2 and *p* < 0.05 was deemed differentially expressed. Median‐of‐ratios for each gene was determined as a raw count of the gene divided by the row‐wise geometric mean to yield a ratio and a median of ratios for all genes in each sample, thus producing a normalization factor for the sample. After normalized counts were calculated for each gene in each sample, a generalized linear model (GLM) with a logarithmic link was fit in order to test for treatment effects (alcohol vs. control) and conditional effects (sex), which returned the coefficients indicating overall expression strength of a gene and log2(fold change) between the treatment groups. After GLMs were fit for each gene, DESeq2 utilized a Wald test for significance (to test the null hypothesis that the logarithmic fold change between the treatment and control group is exactly zero for a given gene's expression), and the resulting Wald test *p* values of a subset of genes that pass independent filtering were adjusted for multiple testing using the Benjamini–Hochberg procedure. During independent filtering, DESeq2 used the average expression strength of each gene, across all samples, as its filter criteria, and omitted all genes with mean normalized counts below a filtering threshold from multiple testing adjustments. By default, DESeq2 chose a threshold that maximized the number of genes found at a user‐specified target false discovery rate (FDR; 0.05). Genesets that satisfied log2 (fold change) ≥2.0 and an FDR adjusted *P*‐value <0.05 were considered differentially expressed.

## 3 RESULTS

**Figure 1:**
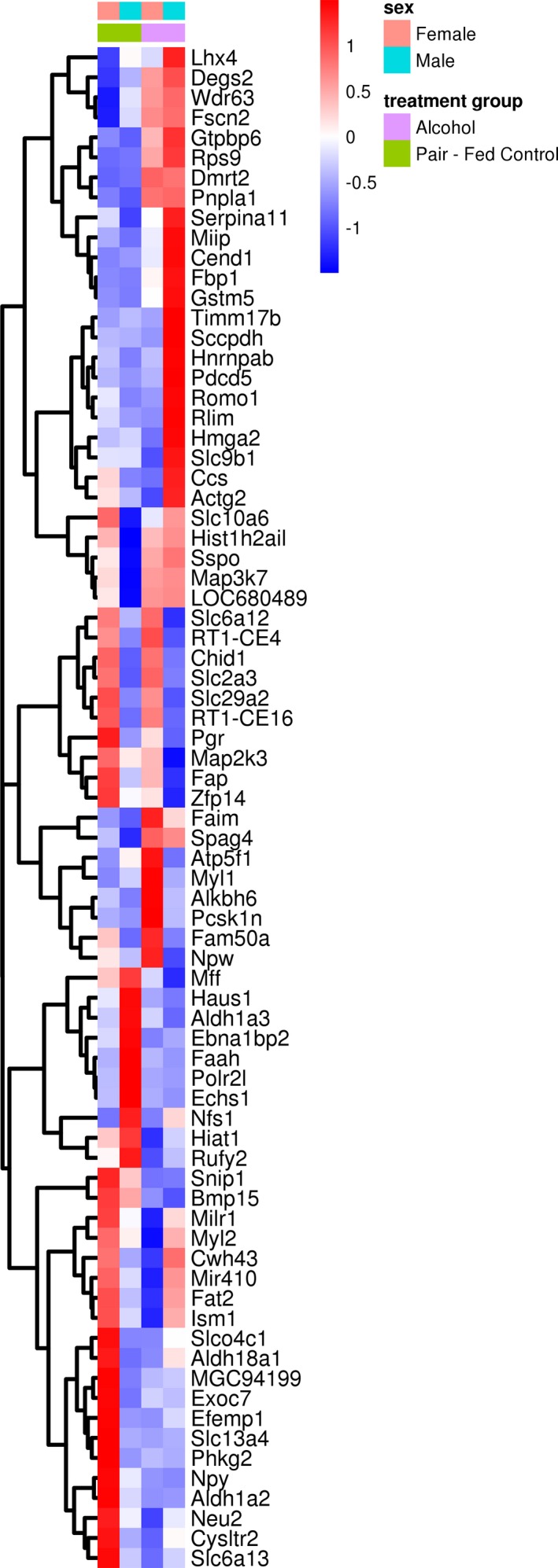
Heat map of RNA‐Seq transcriptome analysis of significantly altered hippocampal genes following our chronic binge prenatal alcohol paradigm. Heat map representation of 76 differentially expressed genes in the fetal hippocampus between pair‐fed Control and alcohol treatment groups, with 37 genes exhibiting downregulation and 39 genes exhibiting upregulation. Map was constructed from the normalized and log‐transformed expression values and subtracted from the row means for each treatment group (*p* < 0.05, and log2 (fold change) ≥2.0)

High‐throughput RNA deep‐sequencing analysis identified 13,388 hippocampal genes, of which 76 showed significant dysregulation following chronic binge gestational alcohol exposure (*p* < 0.05; log2(fold change) ≥2.0). Of these dysregulated genes, 37 exhibited downregulation and 39 expressed upregulation. A heat map illustrates these alterations (Figure 1); expression values based on Pearson correlation values determined the hierarchical clustering structure. Within this group of dysregulated genes, a subset of 49 genes showed sex‐dependent expression differences (*p* < 0.05; log2(fold change) ≥2.0), with 23 genes in alcohol‐exposed females and 26 genes in alcohol‐exposed males showing expression differences when compared to respective PF offspring. Two genes, ATP synthase F1 subunit (*Atp5f1*) and Smad nuclear interacting protein 1 (*Snip1*) exhibited significant dysregulation in both alcohol‐exposed females and males. Interestingly, *Atp5f1* expression increased in ALC females but decreased in ALC males. *Snip1* expression decreased in ALC female and male offspring.

**Figure 2:**
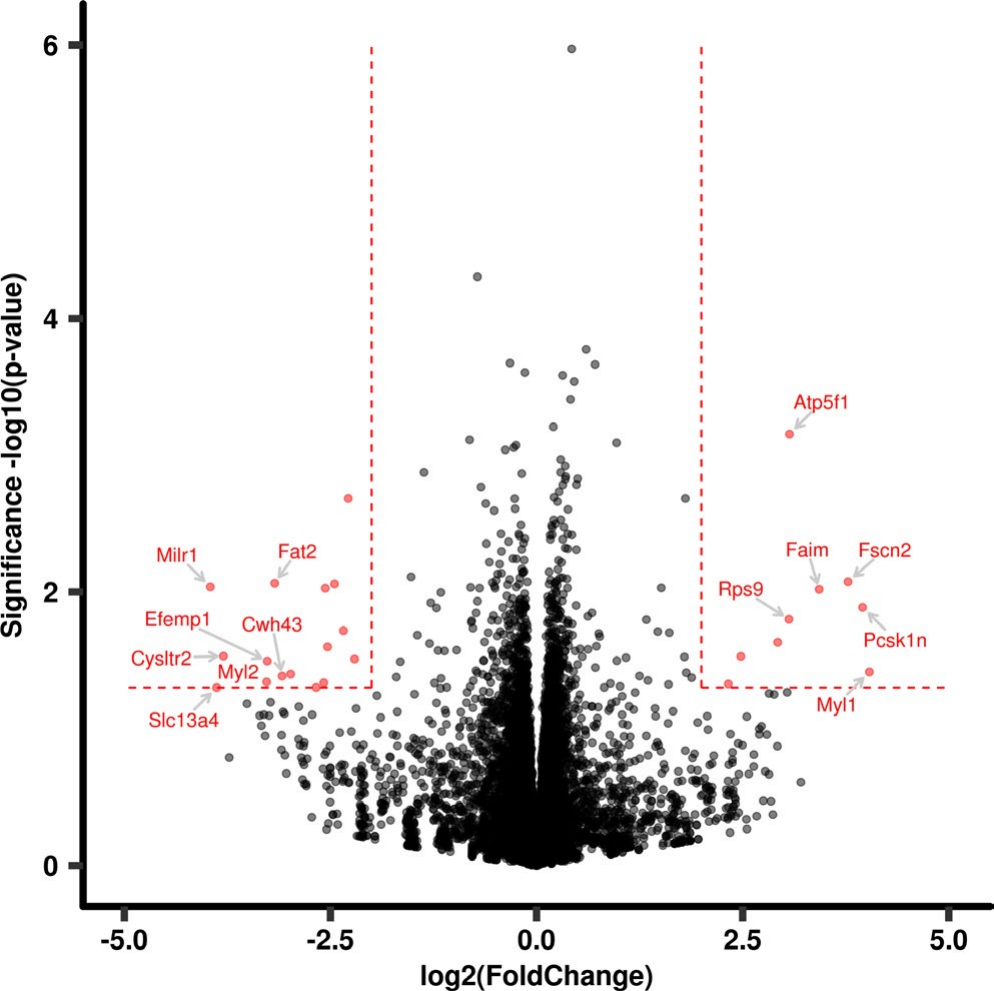
Volcano plot representation of female hippocampal gene expression between the pair‐fed control and alcohol groups. In alcohol‐exposed females, 25 hippocampal genes exhibited sex‐specific alcohol‐induced dysregulation, of which nine were upregulated and 16 were downregulated. Dotted lines denote selection criteria for significance (*p* < 0.05, and log2 (fold change) ≥ 2) and separate differentially expressed genes and similarly expressed genes

Among the 26 hippocampal genes exhibiting expression changes in ALC females, nine displayed upregulation and 16 displayed downregulation (Figure 2). Two downregulated genes, aldehyde dehydrogenase 18 family, member A1 (*Aldh18a1*, ↓) and microRNA 410 (*Mir410*, ↓), have gene‐chemical interactions with choline, an essential nutrient and methyl donor which has been shown to be dysregulated following developmental alcohol exposure and is critically implicated in hippocampal‐based learning tasks (Monk, Leslie, & Thomas, 2012; Ryan et al., 2008). Two other downregulated genes, myosin light chain 2 (*Myl2*, ↓) and phosphorylase kinase catalytic subunit gamma 2 (*Phkg2*, ↓), have gene‐chemical interactions with ethanol. Proprotein convertase subtilisin/kexin type 1 inhibitor (*Pcsk1*, ↑) has previously been identified as involved in brain development, and is also implicated in neuroendocrine signaling (Demoures, Siegfried, & Khatib, 2018). Other genes of interest include neuraminidase 2 (*Neu2*, ↓), for which response to ethanol is a biological process and solute carrier family 6 member 13 (*Slc6a13*, ↓) which is involved in neurotransmitter transport and binding.

**Figure 3:**
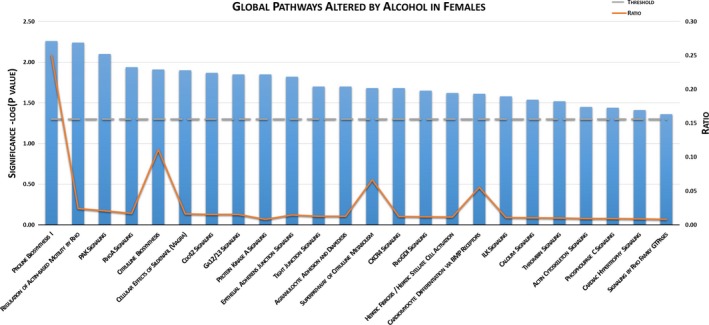
INGENUITY^®^ Pathway Analysis of female hippocampal differentially expressed genes. In alcohol‐exposed females, 24 global pathways were altered compared to the pair‐fed control group (*p* < 0.05). Ratio represents the number of molecules affected to total number of molecules in each pathway

Among female offspring, Bioinformatic INGENUITY^®^ Pathway Analysis (IPA^®^; Figure 3) identified dysregulation of 24 global biological pathways involving differential expression of hippocampal genes following chronic binge gestational alcohol exposure. IPA^®^ determined the top canonical pathways dysregulated in ALC female hippocampi were proline biosynthesis I (*p* = 0.0056), regulation of Actin‐based motility by Rho (*p* = 0.0058), PAK signaling (*p* = 0.0079), RhoA signaling (*p* = 0.011), and citrulline biosynthesis (*p* = 0.012).

**Figure 4:**
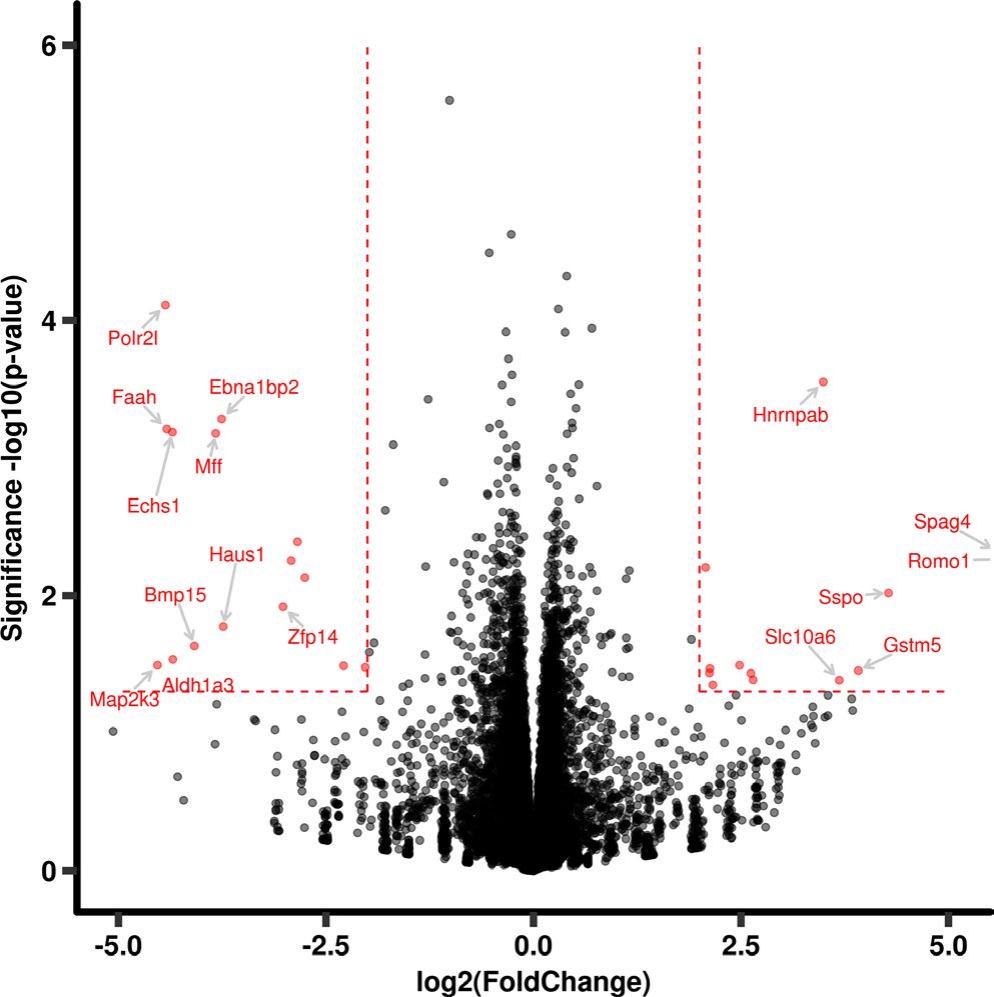
Volcano plot representation of male hippocampal gene expression between the pair‐fed control and alcohol groups. In alcohol‐exposed males, 28 hippocampal genes exhibited sex‐specific alcohol‐induced dysregulation, of which 12 genes were upregulated and 16 genes were downregulated. Dotted lines denote selection criteria for significance (*p* < 0.05, and log2 (fold change) ≥2) and separate differentially expressed genes and similarly expressed genes

Of the 28 male hippocampal genes exhibiting major changes described above, 12 genes showed upregulation and 16 exhibited downregulation (Figure 4). Seven of these dysregulated genes have a known gene‐chemical interaction with choline: Aldehyde dehydrogenase 1 Family Member A3 (*Aldh1a3*, ↓), glutathione S‐transferase, mu 5 (*Gstm5* ↑), programmed cell death 5 (*Pdcd5*, ↑), RUN and FYVE domain containing 2 (*Rufy2*, ↓), saccharopine dehydrogenase (putative) (*Sccpdh*, ↑), sperm‐associated antigen 4 (*Spag4*, ↑), SCO‐spondin (*Sspo*, ↑), zinc finger protein 14 (*Zfp14*, ↓). *Gstm5* (↑) and mitogen‐activated protein kinase kinase 3 (*Map2k3*, ↓) have known gene‐chemical interactions with ethanol. Other genes of interest include *Sspo*, involved in cell differentiation and nervous system development; reactive oxygen species modulator 1 (*Romo1*, ↑), involved in the response to reactive oxygen species, and mitochondrial fission factor (*Mff*, ↓), disease annotations which include developmental disabilities and mitochondrial encephalomyopathy.

**Figure 5:**
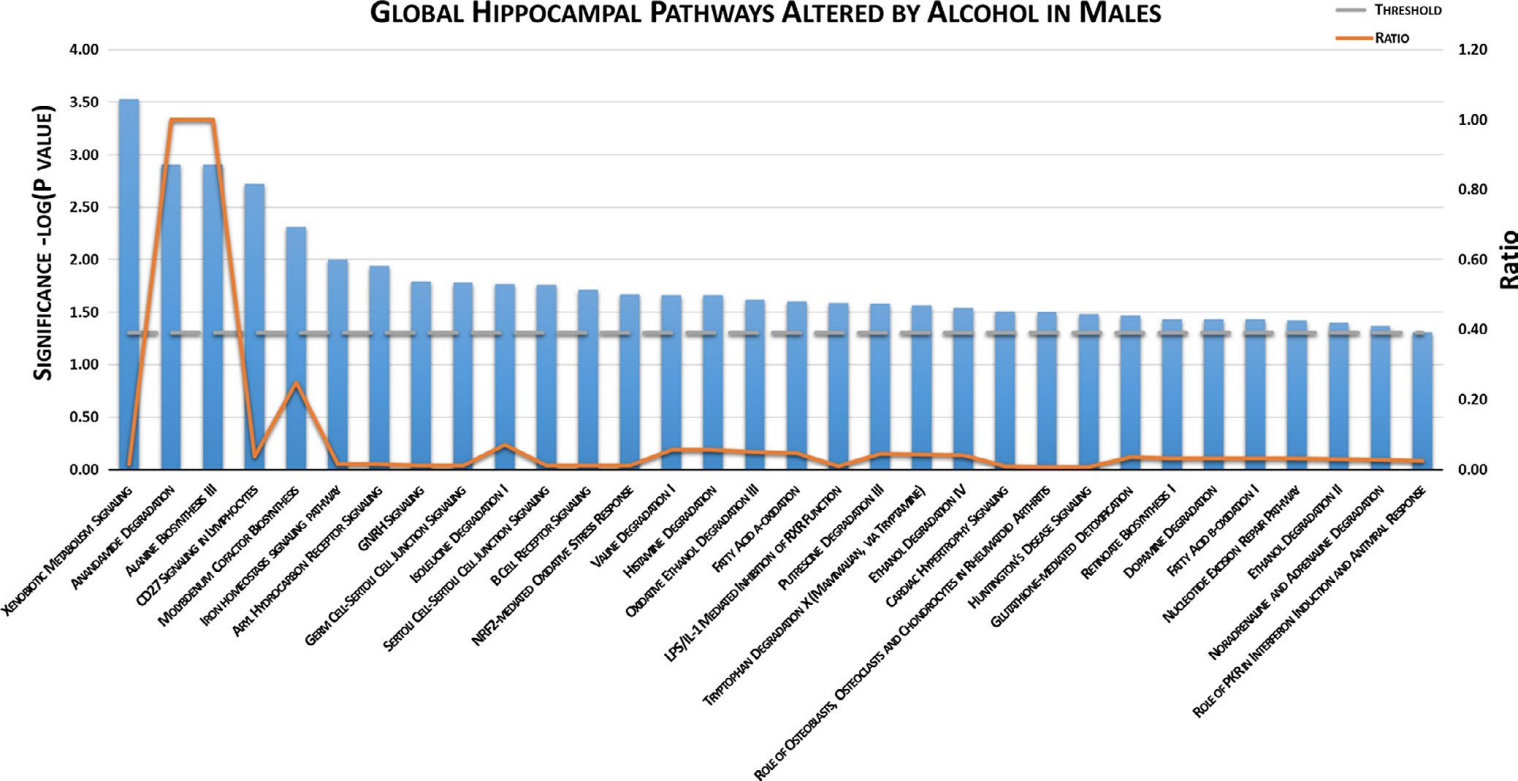
INGENUITY^®^ Pathway Analysis of male hippocampal differentially expressed genes. In alcohol‐exposed males, 32 global pathways were altered compared to the pair‐fed control group (*p* < 0.05). Ratio represents the number of molecules affected to total number of molecules in each pathway

Among male offspring, IPA^®^ (Figure 5) identified dysregulation of 32 global biological pathways involving differential expression of hippocampal genes following chronic binge gestational alcohol exposure. IPA^®^ determined the top canonical pathways dysregulated in ALC male hippocampi were xenobiotic metabolism signaling (*p* = 0.0003), anandamide degradation (*p* = 0.0012), alanine biosynthesis III (*p* = 0.0012), CD27 signaling in lymphocytes (*p* = 0.0019), and molybdenum cofactor biosynthesis (*p* = 0.0049).

## 4 DISCUSSION

To our knowledge, this is the first investigation of the fetal hippocampal transcriptome utilizing next‐gen high‐throughput RNA‐seq following chronic binge gestational alcohol exposure. Three salient findings can be gleaned from this study: (a) a chronic binge paradigm of gestational alcohol exposure dysregulates hippocampal gene expression, (b) this gene dysregulation manifests differently between male and female hippocampi, and (c) gene disruption following our exposure paradigm implicates key global pathways essential for healthy fetal development. Collectively, high‐throughput RNA deep‐sequencing identified 76 hippocampal genes with a significant expression difference between the PF and alcohol‐treated groups, and within this group, a subset of 49 of these genes exhibited sex‐dependent dysregulation.

Among female hippocampi, IPA^®^ determined alcohol dysregulated 24 global canonical pathways following our chronic binge exposure, and includes the following pathways of interest: proline biosynthesis I, citrulline biosynthesis, and the superpathway of citrulline metabolism. *Aldh18a1* encodes for the catalytic enzyme delta‐1‐pyrroline‐5‐carboxylate synthetase (P5CS), which is critical for de novo proline synthesis. Emerging data implicate proline's critical role as a neuroprotectant (Andrade et al., 2018; Sareddy et al., 2015) through opposition to intracellular accumulation of reactive oxygen species (Delwing, Delwing, Chiarani, Kurek, & Wyse, 2007; Krishnan, Dickman, & Becker, 2008), which has been extensively documented as a response to alcohol exposure in the developing brain. Taken in conjunction with proline's established roles as an antagonist to abiotic stressors (Dall'Asta et al., 1999; Ignatova & Gierasch, 2006; Wondrak, Jacobson, & Jacobson, 2005) and an apoptotic regulator (Liu, Borchert, Surazynski, Hu, & Phang, 2006; Rivera & Maxwell, 2005), alcohol‐induced dysregulation of proline biosynthesis I may contribute to alcohol's pathogenesis in the developing hippocampus. Interestingly, *Aldh18a1* is also critically implicated in citrulline biosynthesis and the superpathway of citrulline metabolism. Citrulline biosynthesis occurs downstream from the amino acid precursors glutamate, proline, and arginine, and as arginine is converted to citrulline, nitric oxide (NO) is produced. Interestingly, NO is essential for healthy physiological nervous system regulation and has been shown to have critical roles in synaptic plasticity, learning, and memory (Feil & Kleppisch, 2008; Susswein, Katzoff, Miller, & Hurwitz, 2004). It is possible that alcohol‐induced dysregulation of citrulline‐related biochemical pathways observed in the female hippocampus is reflective of dysregulation of nitric oxide synthase (NOS) activity in this region. *Aldh18a1* downregulation among females may lead to accumulation of its substrate, glutamate, implicating a role for amino acid homeostasis in female hippocampal FASD pathogenesis. Although Aldh18a1 dysregulation has been linked with learning disabilities and neurodevelopmental deficits, hippocampal dysregulation in the context of FASD remains unknown.

Other genes of interest dysregulated by alcohol among female hippocampi include *Mir410*, *Myl2, Phkg2, Pcsk1, and Slc6a13*. Two downregulated genes, *Myl2* and *Phkg2*, have gene‐chemical interactions with ethanol, but to our knowledge have not been previously linked with FASD. *Phkg2*, a regulator of neural and hormonal regulation of glycogen breakdown, has been shown to be downregulated in whole‐brain analysis following prenatal alcohol exposure, but has not been localized to the hippocampus in FASD (Laufer, 2016). *Pcsk1*, a highly expressed gene in the hippocampus that encodes for a serine protease responsible for processing neuropeptides and prohormones, has previously been identified as involved in brain development and is implicated in neuroendocrine signaling (Demoures et al., 2018). In Alzheimer's patients with severe neurodegeneration, the hippocampus is the most vulnerable region of *Pcsk1* dysregulation (Hokama et al., 2013). *Neu2*, for which response to ethanol is a biological process, has shown dysregulation in human embryonic stem cells exposed to alcohol (Khalid et al., 2014) and has been shown to be dysregulated in humans with alcohol dependence (Lingjun et al., 2015). *Slc6a13* (solute carrier family 6 member 13), which is involved in neurotransmitter transport and binding, has also been linked by multiple reports with alcohol use disorders (Hagerty, Bidwell, Harlaar, & Hutchison, 2016; McClintick et al., 2015, 2016), but to our knowledge, its relationship to FASD has not been explored.

Among male hippocampi, IPA^®^ determined alcohol dysregulated 31 global pathways following our chronic binge exposure. Pathways of interest include xenobiotic metabolism signaling, anandamide degradation, alanine biosynthesis III, and molybdenum cofactor biosynthesis. Xenobiotic metabolism signaling describes a cellular stress response to xenobiotic exposure and a concomitant metabolism response to detoxify drugs and other organic compounds (Omiecinski, Vanden Heuvel, Perdew, & Peters, 2010). Genes that were differentially expressed by alcohol and that are associated with this pathway include *Aldh1a3, Gstm3, Map3k7* *, Map2k3*. Interestingly, *Gstm3* and *Map2k3* have known gene‐chemical interactions with ethanol. *Gstm3* is a major detoxification enzyme shown to play a role in the breakdown of xenobiotics including a wide array of drugs and genetic variation is reported to influence susceptibility to toxins (Dasari et al., 2018; Mei et al., 2008). Recent microarray analysis reported dysregulation of glutathione pathways in the synaptoneurosome transcriptome of the mouse amygdala following a chronic alcohol exposure (Most, Ferguson, Blednov, Mayfield, & Harris, 2015). Anandamide is an endogenous neurotransmitter and *Faah,* a key gene within this pathway chiefly responsible for enzymatic breakdown of anandamide, was dysregulated by alcohol. Anandamide dysregulation is associated with hippocampal‐based memory in rats and has been previously speculated to underlie FASD behavioral pathology (Basavarajappa, 2015; Mallet & Beninger, 1996). Molybdenum cofactor biosynthesis and alanine biosynthesis III are directly related, and cysteine desulfurase (*Nfs1)* is implicated in each. Xanthine oxidoreductases are a class of molybdenum cofactor enzymes implicated in cellular responses to senescence and apoptosis (Garattini, Mendel, Romão, Wright, & Terao, 2003), and the conversion of cysteine to alanine (alanine biosynthesis III) through sulfuration of xanthine oxidoreductase renders this class of enzymes catalytically active (Schwarz, 2005). In humans, dysregulation of this process is associated with progressive neurological damage (Johnson, 2001). Collectively, these pathways and their associated genes previously implicated in critical neurodevelopmental processes may play a role in FASD hippocampal pathogenesis observed in male offspring.

Other genes of interest dysregulated among males include: *Sspo*, involved in cell differentiation and nervous system development and has previously been identified as differentially expressed in autism spectrum disorders, bipolar disorder, and schizophrenia (Kember et al., 2015; Krumm et al., 2015; Takata, Ionita‐Laza, Gogos, Xu, & Karayiorgou, 2016); *Romo1*, involved in the response to reactive oxygen species and TNF‐induced apoptosis (Bae, Oh, Rhee, & Do Yoo, 2011; Lee et al., 2010; Redza‐Dutordoir & Averill‐Bates, 2016); and *Mff*, which is essential for embryonic development and synapse formation disease and annotations for which it include developmental disabilities and mitochondrial encephalomyopathy (Ishihara et al., 2009). Seven dysregulated genes (*Aldh1a3, Gstm5, Pdcd5, Rufy2, Sccpdh, Spag4,* and *Sspo*) have a known gene‐chemical interaction with choline. Prenatal alcohol‐induced dysregulation of choline bioavailability is associated with impaired hippocampal development, learning, and memory (Niculescu, Craciunescu, & Zeisel, 2006), and we conjecture that these choline‐interacting genes play an underlying role in this established alcohol‐induced neuropathology. Though these genes have been previously implicated in alcohol‐related neurological dysfunction, their roles in FASD hippocampal deficits remain to be explored.

Sex‐based differences identified in the brain, and specifically in the hippocampus, have been shown to differentially affect susceptibility to disease, neurological function, and behaviors (Ngun, Ghahramani, Sánchez, Bocklandt, & Vilain, 2011). Collaborative reports investigating FASD models have implicated abundant alcohol‐induced sex‐specific hippocampal effects. Hippocampal neuroimmune response measured in offspring on PND 5 and 8 demonstrated a sex‐dependent response to a developmental alcohol challenge (Ruggiero, Boschen, Roth, & Klintsova, 2018). Adolescent hippocampal functional assessment revealed N‐methyl‐D‐aspartate long‐term potentiation reduced by 40% in adolescent males prenatally exposed to alcohol compared to adolescent females; interestingly, females exhibited increased hippocampal glutamine synthetase expression (Sickmann et al., 2014). Prenatal alcohol exposure has also been shown to have sex‐specific hippocampal effects lasting into adulthood, as Uban and colleagues demonstrated that PND 60 females exhibit a reduced proportion of newly produced neurons and glia in the dentate gyrus compared with males (Uban et al., 2010). These studies indicate that alcohol has the potential to affect this brain region differentially based on sex, however, a knowledge gap persists regarding differences in hippocampal gene expression profiles between male and female rats alone and even more in the context of FASD (Schneider, Anderson, Sonnenahalli, & Vadigepalli, 2011; Tunc‐Ozcan et al., 2013; Van den Hove et al., 2013). An understanding of these differential outcomes at the transcriptome level is fundamental for developing novel therapeutic strategies that account for these sex‐based differences for maximum effectiveness.

### 4.1 Perspectives and significance

Thus far, the effects of chronic binge gestational alcohol exposure on hippocampal transcriptome‐wide gene expression have remained largely limited to microarray analyses. The majority of hippocampal microarray analyses in animal models of FASD has been performed on: (a) adolescent or adult animals exposed to alcohol during development (Chater‐Diehl et al., 2016; Lussier et al., 2015; Marjonen et al., 2015), or (b) on animals whose exposure paradigm did not mimic a chronic binge exposure paradigm throughout pregnancy (Mandal et al., 2015). To our knowledge, no microarray has analyzed rat hippocampal gene expression using a chronic binge model of gestational alcohol exposure. By utilizing next‐gen high‐throughput RNA‐seq, our goal was to elucidate the novel molecular targets underlying FASD hippocampal deficits to better understand FASD pathogenesis. In summary, our results indicate that a chronic binge paradigm of gestational alcohol exposure differentially alters the hippocampal gene expression, and that this alcohol‐induced gene expression exhibits sex‐specific variation in the developing hippocampus, as do their associated global canonical pathways. Detection of subtle gene expression changes within specific brain regions, such as the hippocampus, through advances in next‐generation sequencing may yield critical new understanding of vulnerable genes and genetic networks underlying FASD neuropathogenesis. Insights acquired from this advanced genomic technology offer novel findings essential for pinpointing targets of developmental alcohol exposure necessary for the development of urgently needed, targeted therapeutic intervention strategies.

## CONFLICT OF INTEREST

None.

## Data Availability

The data that support the findings will be available in U.S. National Library of Medicine, NCBI Sequence Read Archive (SRA) following an embargo that ends on 14 February 2020 (Ramadoss, 2019).

## References

[brb31334-bib-0001] Andrade, V. S. , Rojas, D. B. , de Andrade, R. B. , Kim, T. D. H. , Vizuete, A. F. , Zanatta, Â. , … Wannmacher, C. M. D. (2018). A possible anti‐inflammatory effect of proline in the brain cortex and cerebellum of rats. Molecular Neurobiology, 55(5), 4068–4077. 28585188 10.1007/s12035-017-0626-z

[brb31334-bib-0002] Autti‐Rämö, I. , Autti, T. , Korkman, M. , Kettunen, S. , Salonen, O. , & Valanne, L. (2002). MRI findings in children with school problems who had been exposed prenatally to alcohol. Developmental Medicine and Child Neurology, 44(2), 98–106. 10.1017/S0012162201001748 11848116

[brb31334-bib-0003] Bae, Y. S. , Oh, H. , Rhee, S. G. , & Do Yoo, Y. (2011). Regulation of reactive oxygen species generation in cell signaling. Molecules and Cells, 32(6), 491–509. 10.1007/s10059-011-0276-3 22207195 PMC3887685

[brb31334-bib-0004] Basavarajappa, B. (2015). Fetal alcohol spectrum disorder: Potential role of endocannabinoids signaling. Brain Sciences, 5(4), 456–493. 10.3390/brainsci5040456 26529026 PMC4701023

[brb31334-bib-0005] Berman, R. F. , & Hannigan, J. H. (2000). Effects of prenatal alcohol exposure on the hippocampus: Spatial behavior, electrophysiology, and neuroanatomy. Hippocampus, 10(1), 94–110. 10.1002/(SICI)1098-1063(2000)10:1<94:AID-HIPO11>3.0.CO;2-T 10706221

[brb31334-bib-0006] Bhattacharya, D. , Dunaway, E. P. , Bhattacharya, S. , Bloemer, J. , Buabeid, M. , Escobar, M. , … Dhanasekaran, M. (2015). Impaired ILK function is associated with deficits in hippocampal based memory and synaptic plasticity in a FASD rat model. PLoS ONE, 10(8), e0135700 10.1371/journal.pone.0135700 26305322 PMC4549293

[brb31334-bib-0007] Bolger, A. M. , Lohse, M. , & Usadel, B. (2014). Trimmomatic: A flexible trimmer for Illumina sequence data. Bioinformatics, 30(15), 2114–2120. 10.1093/bioinformatics/btu170 24695404 PMC4103590

[brb31334-bib-0008] Boschen, K. E. , & Klintsova, A. Y. (2017). Disruptions to hippocampal adult neurogenesis in rodent models of fetal alcohol spectrum disorders. Neurogenesis, 4(1), e1324259 10.1080/23262133.2017.1324259

[brb31334-bib-0009] Burd, L. (2016). Fetal alcohol spectrum disorder: Complexity from comorbidity. The Lancet, 387(10022), 926–927. 10.1016/S0140-6736(15)01346-X 26777271

[brb31334-bib-0010] Caetano, R. , Ramisetty-Mikler, S. , Floyd, L.R. & McGrath, C. (2006). The epidemiology of drinking among women of child-bearing age. Alcoholism: Clinical and Experimental Research, 30(6), 1023–1030. 10.1111/j.1530-0277.2006.00116.x 16737461

[brb31334-bib-0011] Caputo, C. , Wood, E. , & Jabbour, L. (2016). Impact of fetal alcohol exposure on body systems: A systematic review. Birth Defects Research Part C: Embryo Today: Reviews, 108(2), 174–180. 10.1002/bdrc.21129 27297122

[brb31334-bib-0012] Chater‐Diehl, E. J. , Laufer, B. I. , Castellani, C. A. , Alberry, B. L. , & Singh, S. M. (2016). Alteration of gene expression, DNA methylation, and histone methylation in free radical scavenging networks in adult mouse hippocampus following fetal alcohol exposure. PLoS ONE, 11(5), e0154836 10.1371/journal.pone.0154836 27136348 PMC4852908

[brb31334-bib-0013] Church, M. W. , & Gerkin, K. P. (1988). Hearing disorders in children with fetal alcohol syndrome: findings from case reports. Pediatrics, 82(2), 147–154. 3399287

[brb31334-bib-0014] Cudd, T.A. , Chen, W.J.A. & West, J.R. (2002). Fetal and maternal thyroid hormone responses to ethanol exposure during the third trimester equivalent of gestation in sheep. Alcoholism: Clinical and Experimental Research, 26(1), 53–58. 11821654

[brb31334-bib-0015] Dall'Asta, V. , Bussolati, O. , Sala, R. , Parolari, A. , Alamanni, F. , Biglioli, P. , & Gazzola, G. C. (1999). Amino acids are compatible osmolytes for volume recovery after hypertonic shrinkage in vascular endothelial cells. American Journal of Physiology‐Cell Physiology, 276(4), C865–C872. 10.1152/ajpcell.1999.276.4.C865 10199817

[brb31334-bib-0016] Dasari, S. , Gonuguntla, S. , Ganjayi, M. S. , Bukke, S. , Sreenivasulu, B. , & Meriga, B. (2018). Genetic polymorphism of glutathione S‐transferases: Relevance to neurological disorders. Pathophysiology, 25(4), 285–292. 10.1016/j.pathophys.2018.06.001 29908890

[brb31334-bib-0017] Davis‐Anderson, K. L. , Wesseling, H. , Siebert, L. M. , Lunde‐Young, E. R. , Naik, V. D. , Steen, H. , & Ramadoss, J. (2018). Fetal regional brain protein signature in FASD rat model. Reproductive Toxicology, 76, 84–92. 10.1016/j.reprotox.2018.01.004 29408587 PMC5834402

[brb31334-bib-0018] Delwing, D. , Delwing, D. , Chiarani, F. , Kurek, A. G. , & Wyse, A. T. (2007). Proline reduces brain cytochrome c oxidase: Prevention by antioxidants. International Journal of Developmental Neuroscience, 25(1), 17–22. 10.1016/j.ijdevneu.2006.11.005 17197150

[brb31334-bib-0019] Demoures, B. , Siegfried, G. , & Khatib, A.‐M. (2018). PCSK1 (proprotein convertase subtilisin/kexin type 1). Atlas of Genetics and Cytogenetics in Oncology and Haematology, 6, 10.4267/2042/68910

[brb31334-bib-0020] Dudek, J. , Skocic, J. , Sheard, E. , & Rovet, J. (2014). Hippocampal abnormalities in youth with alcohol‐related neurodevelopmental disorder. Journal of the International Neuropsychological Society, 20(2), 181–191. 10.1017/S1355617713001343 24512673

[brb31334-bib-0021] Ethen, M. K. , Ramadhani, T. A. , Scheuerle, A. E. , Canfield, M. A. , Wyszynski, D. F. , Druschel, C. M. , & Romitti, P. A. (2009). Alcohol consumption by women before and during pregnancy. Maternal and Child Health Journal, 13(2), 274–285. 10.1007/s10995-008-0328-2 18317893 PMC6090563

[brb31334-bib-0022] Feil, R. , & Kleppisch, T. (2008). NO/cGMP‐dependent modulation of synaptic transmission In Südhof T. C., Starke K. (Eds.), Pharmacology of Neurotransmitter Release (pp. 529–560). Springer. 10.1007/978-3-540-74805-2_16 18064424

[brb31334-bib-0023] Fontaine, C. J. , Patten, A. R. , Sickmann, H. M. , Helfer, J. L. , & Christie, B. R. (2016). Effects of pre‐natal alcohol exposure on hippocampal synaptic plasticity: Sex, age and methodological considerations. Neuroscience & Biobehavioral Reviews, 64, 12–34. 10.1016/j.neubiorev.2016.02.014 26906760

[brb31334-bib-0024] Garattini, E. , Mendel, R. , Romão, M. J. , Wright, R. , & Terao, M. (2003). Mammalian molybdo‐flavoenzymes, an expanding family of proteins: Structure, genetics, regulation, function and pathophysiology. Biochemical Journal, 372(1), 15–32. 10.1042/bj20030121 12578558 PMC1223366

[brb31334-bib-0025] Gil‐Mohapel, J. , Boehme, F. , Patten, A. , Cox, A. , Kainer, L. , Giles, E. , … Christie, B. R. (2011). Altered adult hippocampal neuronal maturation in a rat model of fetal alcohol syndrome. Brain Research, 1384, 29–41. 10.1016/j.brainres.2011.01.116 21303667

[brb31334-bib-0026] Hagerty, S. L. , Bidwell, L. C. , Harlaar, N. , & Hutchison, K. E. (2016). An exploratory association study of alcohol use disorder and DNA methylation. Alcoholism: Clinical and Experimental Research, 40(8), 1633–1640. 10.1111/acer.13138 PMC5108727 27388583

[brb31334-bib-0027] Hamilton, D. A. , Kodituwakku, P. , Sutherland, R. J. , & Savage, D. D. (2003). Children with Fetal Alcohol Syndrome are impaired at place learning but not cued‐navigation in a virtual Morris water task. Behavioural Brain Research, 143(1), 85–94. 10.1016/S0166-4328(03)00028-7 12842299

[brb31334-bib-0028] Hofer, R. , & Burd, L. (2009). Review of published studies of kidney, liver, and gastrointestinal birth defects in fetal alcohol spectrum disorders. Birth Defects Research Part A: Clinical and Molecular Teratology, 85(3), 179–183. 10.1002/bdra.20562 19180632

[brb31334-bib-0029] Hokama, M. , Oka, S. , Leon, J. , Ninomiya, T. , Honda, H. , Sasaki, K. , … Nakabeppu, Y. (2013). Altered expression of diabetes‐related genes in Alzheimer's disease brains: The Hisayama study. Cerebral Cortex, 24(9), 2476–2488. 10.1093/cercor/bht101 23595620 PMC4128707

[brb31334-bib-0030] Ignatova, Z. , & Gierasch, L. M. (2006). Inhibition of protein aggregation in vitro and in vivo by a natural osmoprotectant. Proceedings of the National Academy of Sciences, 103(36), 13357–13361. 10.1073/pnas.0603772103 PMC1569168 16899544

[brb31334-bib-0031] Ishihara, N. , Nomura, M. , Jofuku, A. , Kato, H. , Suzuki, S. O. , Masuda, K. , … Mihara, K. (2009). Mitochondrial fission factor Drp1 is essential for embryonic development and synapse formation in mice. Nature Cell Biology, 11(8), 958 10.1038/ncb1907 19578372

[brb31334-bib-0032] Johnson, J. (2001). Molybdenum cofactor deficiency and isolated sulfite oxidase deficiency. The Metabolic and Molecular Bases of Inherited Disease, 3163–3177.

[brb31334-bib-0033] Kajimoto, K. , Valenzuela, C. F. , Allan, A. M. , Ge, S. , Gu, Y. , & Cunningham, L. A. (2016). Prenatal alcohol exposure alters synaptic activity of adult hippocampal dentate granule cells under conditions of enriched environment. Hippocampus, 26(8), 1078–1087. 10.1002/hipo.22588 27009742 PMC4949153

[brb31334-bib-0034] Kember, R. L. , Georgi, B. , Bailey‐Wilson, J. E. , Stambolian, D. , Paul, S. M. , & Bućan, M. (2015). Copy number variants encompassing Mendelian disease genes in a large multigenerational family segregating bipolar disorder. BMC Genetics, 16(1), 27 10.1186/s12863-015-0184-1 25887117 PMC4382929

[brb31334-bib-0035] Khalid, O. , Kim, J. J. , Kim, H.‐S. , Hoang, M. , Tu, T. G. , Elie, O. , … Kim, Y. (2014). Gene expression signatures affected by alcohol‐induced DNA methylomic deregulation in human embryonic stem cells. Stem Cell Research, 12(3), 791–806. 10.1016/j.scr.2014.03.009 24751885 PMC4041389

[brb31334-bib-0036] Kim, D. , Langmead, B. , & Salzberg, S. L. (2015). HISAT: A fast spliced aligner with low memory requirements. Nature Methods, 12(4), 357 10.1038/nmeth.3317 25751142 PMC4655817

[brb31334-bib-0037] Krishnan, N. , Dickman, M. B. , & Becker, D. F. (2008). Proline modulates the intracellular redox environment and protects mammalian cells against oxidative stress. Free Radical Biology and Medicine, 44(4), 671–681. 10.1016/j.freeradbiomed.2007.10.054 18036351 PMC2268104

[brb31334-bib-0038] Krumm, N. , Turner, T. N. , Baker, C. , Vives, L. , Mohajeri, K. , Witherspoon, K. , … Eichler, E. E. (2015). Excess of rare, inherited truncating mutations in autism. Nature Genetics, 47(6), 582 10.1038/ng.3303 25961944 PMC4449286

[brb31334-bib-0039] Laufer, B. I. (2016). A long‐term neuroepigenomic profile of prenatal alcohol exposure. Electronic Thesis and Dissertation Repository. 3913. https://ir.lib.uwo.ca/etd/3913

[brb31334-bib-0040] Lee, S. B. , Kim, J. J. , Kim, T. W. , Kim, B. S. , Lee, M.‐S. , & Do Yoo, Y. (2010). Serum deprivation‐induced reactive oxygen species production is mediated by Romo1. Apoptosis, 15(2), 204–218. 10.1007/s10495-009-0411-1 19904609

[brb31334-bib-0041] Lewis, C. E. , Thomas, K. G. , Dodge, N. C. , Molteno, C. D. , Meintjes, E. M. , Jacobson, J. L. , & Jacobson, S. W. (2015). Verbal learning and memory impairment in children with fetal alcohol spectrum disorders. Alcoholism: Clinical and Experimental Research, 39(4), 724–732. 10.1111/acer.12671 PMC4384160 25833031

[brb31334-bib-0042] Liao, Y. , Smyth, G. K. , & Shi, W. (2013). featureCounts: An efficient general purpose program for assigning sequence reads to genomic features. Bioinformatics, 30(7), 923–930. 10.1093/bioinformatics/btt656 24227677

[brb31334-bib-0043] Lingjun, Z. , Zhang, C. K. , Sayward, F. G. , Cheung, K.‐H. , Kesheng, W. , Krystal, J. H. , … Xingguang, L. (2015). Gene‐based and pathway‐based genome‐wide association study of alcohol dependence. Shanghai Archives of Psychiatry, 27(2), 111. 26120261 10.11919/j.issn.1002-0829.215031 PMC4466852

[brb31334-bib-0044] Liu, Y. , Borchert, G. , Surazynski, A. , Hu, C. , & Phang, J. (2006). Proline oxidase activates both intrinsic and extrinsic pathways for apoptosis: The role of ROS/superoxides, NFAT and MEK/ERK signaling. Oncogene, 25(41), 5640 10.1038/sj.onc.1209564 16619034

[brb31334-bib-0045] Love, M. I. , Huber, W. , & Anders, S. (2014). Moderated estimation of fold change and dispersion for RNA‐seq data with DESeq2. Genome Biology, 15(12), 550 10.1186/s13059-014-0550-8 25516281 PMC4302049

[brb31334-bib-0046] Lussier, A. A. , Stepien, K. A. , Weinberg, J. , & Kobor, M. S. (2015). Prenatal alcohol exposure alters gene expression in the rat brain: Experimental design and bioinformatic analysis of microarray data. Data in Brief, 4, 239–252. 10.1016/j.dib.2015.05.007 26217797 PMC4510447

[brb31334-bib-0047] Maier, S. E. , & West, J. R. (2001). Drinking patterns and alcohol‐related birth defects. Alcohol Research and Health, 25(3), 168–174. 11810954 PMC6707176

[brb31334-bib-0048] Mallet, P. , & Beninger, R. (1996). The endogenous cannabinoid receptor agonist. Behavioural Pharmacology, 7, 276–284.

[brb31334-bib-0049] Mandal, C. , Park, K. S. , Jung, K. H. , & Chai, Y. G. (2015). Ethanol‐related alterations in gene expression patterns in the developing murine hippocampus. Acta Biochimica Et Biophysica Sinica, 47(8), 581–587. 10.1093/abbs/gmv050 26063602

[brb31334-bib-0050] Mantha, K. , Kleiber, M. , & Singh, S. (2013). Neurodevelopmental timing of ethanol exposure may contribute to observed heterogeneity of behavioral deficits in a mouse model of fetal alcohol spectrum disorder (FASD). Journal of Behavioral and Brain Science, 3(01), 85 10.4236/jbbs.2013.31009

[brb31334-bib-0051] Marjonen, H. , Sierra, A. , Nyman, A. , Rogojin, V. , Gröhn, O. , Linden, A.‐M. , … Kaminen‐Ahola, N. (2015). Early maternal alcohol consumption alters hippocampal DNA methylation, gene expression and volume in a mouse model. PLoS ONE, 10(5), e0124931 10.1371/journal.pone.0124931 25970770 PMC4430308

[brb31334-bib-0052] May, P. A. , Blankenship, J. , Marais, A.‐S. , Gossage, J. P. , Kalberg, W. O. , Joubert, B. , … Seedat, S. (2013). Maternal alcohol consumption producing fetal alcohol spectrum disorders (FASD): Quantity, frequency, and timing of drinking. Drug and Alcohol Dependence, 133(2), 502–512. 10.1016/j.drugalcdep.2013.07.013 23932841 PMC3829200

[brb31334-bib-0053] May, P. A. , Chambers, C. D. , Kalberg, W. O. , Zellner, J. , Feldman, H. , Buckley, D. , … Hoyme, H. E. (2018). Prevalence of fetal alcohol spectrum disorders in 4 US communities. JAMA, 319(5), 474–482. 10.1001/jama.2017.21896 29411031 PMC5839298

[brb31334-bib-0054] May, P. A. , & Gossage, J. P. (2011). Maternal risk factors for fetal alcohol spectrum disorders: Not as simple as it might seem. Alcohol Research & Health, 34(1), 15. 23580036 PMC3860552

[brb31334-bib-0055] McClintick, J. N. , McBride, W. J. , Bell, R. L. , Ding, Z.‐M. , Liu, Y. , Xuei, X. , & Edenberg, H. J. (2015). Gene expression changes in serotonin, GABA‐A receptors, neuropeptides and ion channels in the dorsal raphe nucleus of adolescent alcohol‐preferring (P) rats following binge‐like alcohol drinking. Pharmacology Biochemistry and Behavior, 129, 87–96. 10.1016/j.pbb.2014.12.007 PMC4302739 25542586

[brb31334-bib-0056] McClintick, J. N. , McBride, W. J. , Bell, R. L. , Ding, Z. M. , Liu, Y. , Xuei, X. , & Edenberg, H. J. (2016). Gene expression changes in glutamate and GABA‐A receptors, neuropeptides, ion channels, and cholesterol synthesis in the periaqueductal gray following binge‐like alcohol drinking by adolescent alcohol‐preferring (P) rats. Alcoholism: Clinical and Experimental Research, 40(5), 955–968. 10.1111/acer.13056 PMC4844794 27061086

[brb31334-bib-0057] Mei, N. , Guo, L. , Tseng, J. , Dial, S. L. , Liao, W. , & Manjanatha, M. G. (2008). Gene expression changes associated with xenobiotic metabolism pathways in mice exposed to acrylamide. Environmental and Molecular Mutagenesis, 49(9), 741–745. 10.1002/em.20429 18800343 PMC5739318

[brb31334-bib-0058] Monk, B. R. , Leslie, F. M. , & Thomas, J. D. (2012). The effects of perinatal choline supplementation on hippocampal cholinergic development in rats exposed to alcohol during the brain growth spurt. Hippocampus, 22(8), 1750–1757. 10.1002/hipo.22009 22431326 PMC3382021

[brb31334-bib-0059] Most, D. , Ferguson, L. , Blednov, Y. , Mayfield, R. , & Harris, R. (2015). The synaptoneurosome transcriptome: A model for profiling the emolecular effects of alcohol. The Pharmacogenomics Journal, 15(2), 177 10.1038/tpj.2014.43 25135349 PMC4334750

[brb31334-bib-0060] Ngun, T. C. , Ghahramani, N. , Sánchez, F. J. , Bocklandt, S. , & Vilain, E. (2011). The genetics of sex differences in brain and behavior. Frontiers in Neuroendocrinology, 32(2), 227–246. 10.1016/j.yfrne.2010.10.001 20951723 PMC3030621

[brb31334-bib-0061] Niculescu, M. D. , Craciunescu, C. N. , & Zeisel, S. H. (2006). Dietary choline deficiency alters global and gene‐specific DNA methylation in the developing hippocampus of mouse fetal brains. The FASEB Journal, 20(1), 43–49. 10.1096/fj.05-4707com 16394266 PMC1635129

[brb31334-bib-0062] Omiecinski, C. J. , Vanden Heuvel, J. P. , Perdew, G. H. , & Peters, J. M. (2010). Xenobiotic metabolism, disposition, and regulation by receptors: From biochemical phenomenon to predictors of major toxicities. Toxicological Sciences, 120(suppl_1), S49–S75. 21059794 10.1093/toxsci/kfq338 PMC3145385

[brb31334-bib-0063] Perez, H. D. , Villanueva, J. E. , & Salas, J. M. (1991). Behavioral and hippocampal morphological changes induced by ethanol administered to pregnant rats. Annals of the New York Academy of Sciences, 625(1), 300–304. 10.1111/j.1749-6632.1991.tb33855.x 2058890

[brb31334-bib-0064] Popova, S. , Lange, S. , Shield, K. , Mihic, A. , Chudley, A. E. , Mukherjee, R. A. S. , … Rehm, J. (2016). Comorbidity of fetal alcohol spectrum disorder: A systematic review and meta‐analysis. The Lancet, 387(10022), 978–987. 10.1016/S0140-6736(15)01345-8 26777270

[brb31334-bib-0065] Ramadoss, J. (2019). Fetal hippocampus FASD RNA Sequencing. U.S. National Library of Medicine, NCBI Sequence Read Archive (SRA) SUB5191252.

[brb31334-bib-0066] Ramos, A. J. , Evrard, S. G. , Tagliaferro, P. , TricÁrico, M. V. , & Brusco, A. (2002). Effects of chronic maternal ethanol exposure on hippocampal and striatal morphology in offspring. Annals of the New York Academy of Sciences, 965(1), 343–353. 10.1111/j.1749-6632.2002.tb04176.x 12105110

[brb31334-bib-0067] Redza‐Dutordoir, M. , & Averill‐Bates, D. A. (2016). Activation of apoptosis signalling pathways by reactive oxygen species. Biochimica Et Biophysica Acta (BBA) ‐ Molecular Cell Research, 1863(12), 2977–2992. 10.1016/j.bbamcr.2016.09.012 27646922

[brb31334-bib-0068] Riley, E. P. , Infante, M. A. , & Warren, K. R. (2011). Fetal alcohol spectrum disorders: An overview. Neuropsychology Review, 21(2), 73 10.1007/s11065-011-9166-x 21499711 PMC3779274

[brb31334-bib-0069] Rivera, A. , & Maxwell, S. A. (2005). The p53‐induced gene‐6 (proline oxidase) mediates apoptosis through a calcineurin‐dependent pathway. Journal of Biological Chemistry, 280(32), 29346–29354. 10.1074/jbc.M504852200 15914462

[brb31334-bib-0070] Ruggiero, M. J. , Boschen, K. E. , Roth, T. L. , & Klintsova, A. Y. (2018). Sex Differences in Early Postnatal Microglial Colonization of the Developing Rat Hippocampus Following a Single-Day Alcohol Exposure. Journal of Neuroimmune Pharmacology, 13(2), 89–203. 10.1007/s11481-017-9774-1 PMC5997457 29274031

[brb31334-bib-0071] Ryan, S. H. , Williams, J. K. , & Thomas, J. D. (2008). Choline supplementation attenuates learning deficits associated with neonatal alcohol exposure in the rat: Effects of varying the timing of choline administration. Brain Research, 1237, 91–100. 10.1016/j.brainres.2008.08.048 18786517 PMC2646103

[brb31334-bib-0072] Sareddy, G. R. , Zhang, Q. , Wang, R. , Scott, E. , Zou, Y. I. , O'Connor, J. C. , … Brann, D. (2015). Proline‐, glutamic acid‐, and leucine‐rich protein 1 mediates estrogen rapid signaling and neuroprotection in the brain. Proceedings of the National Academy of Sciences, 112(48), E6673–E6682. 10.1073/pnas.1516729112 PMC4672783 26627258

[brb31334-bib-0073] Schneider, J. , Anderson, D. , Sonnenahalli, H. , & Vadigepalli, R. (2011). Sex‐based differences in gene expression in hippocampus following postnatal lead exposure. Toxicology and Applied Pharmacology, 256(2), 179–190. 10.1016/j.taap.2011.08.008 21864555 PMC3183343

[brb31334-bib-0074] Schwarz, G. (2005). Molybdenum cofactor biosynthesis and deficiency. Cellular and Molecular Life Sciences CMLS, 62(23), 2792–2810. 10.1007/s00018-005-5269-y 16261263 PMC11145942

[brb31334-bib-0075] Smith, S. M. , Garic, A. , Berres, M. E. , & Flentke, G. R. (2014). Genomic factors that shape craniofacial outcome and neural crest vulnerability in FASD. Frontiers in Genetics, 5, 224 10.3389/fgene.2014.00224 25147554 PMC4124534

[brb31334-bib-0076] Sokol, R. J. , Delaney‐Black, V. , & Nordstrom, B. (2003). Fetal alcohol spectrum disorder. JAMA, 290(22), 2996–2999. 10.1001/jama.290.22.2996 14665662

[brb31334-bib-0077] Spohr, H.‐L. , & Steinhausen, H.‐C. (2008). Fetal alcohol spectrum disorders and their persisting sequelae in adult life. Deutsches Ärzteblatt International, 105(41), 693 10.3238/arztebl.2008.0693 19623288 PMC2696967

[brb31334-bib-0078] Susswein, A. J. , Katzoff, A. , Miller, N. , & Hurwitz, I. (2004). Nitric oxide and memory. The Neuroscientist, 10(2), 153–162. 10.1177/1073858403261226 15070489

[brb31334-bib-0079] Takata, A. , Ionita‐Laza, I. , Gogos, J. A. , Xu, B. , & Karayiorgou, M. (2016). De novo synonymous mutations in regulatory elements contribute to the genetic etiology of autism and schizophrenia. Neuron, 89(5), 940–947. 10.1016/j.neuron.2016.02.024 26938441 PMC4793939

[brb31334-bib-0080] Tan, C. H. , Denny, C. H. , Cheal, N. E. , Sniezek, J. E. , & Kanny, D. (2015). Alcohol use and binge drinking among women of childbearing age ‐ United States, 2011–2013. Morbidity and Mortality Weekly Report, 64(37), 1042–1046. 10.15585/mmwr.mm6437a3 26401713

[brb31334-bib-0081] Thanh, N. X. , Jonsson, E. , Dennett, L. , & Jacobs, P. (2011). Costs of FASD. Fetal Alcohol Spectrum Disorder, 4, 1.

[brb31334-bib-0082] Thomas, J. D. , Idrus, N. M. , Monk, B. R. , & Dominguez, H. D. (2010). Prenatal choline supplementation mitigates behavioral alterations associated with prenatal alcohol exposure in rats. Birth Defects Research Part A: Clinical and Molecular Teratology, 88(10), 827–837. 20706995 10.1002/bdra.20713 PMC3677823

[brb31334-bib-0083] Tunc‐Ozcan, E. , Ullmann, T. M. , Shukla, P. K. , & Redei, E. E. (2013). Low‐dose thyroxine attenuates autism‐associated adverse effects of fetal alcohol in male offspring's social behavior and hippocampal gene expression. Alcoholism: Clinical and Experimental Research, 37(11), 1986–1995. 10.1111/acer.12183 PMC3805686 23763370

[brb31334-bib-0084] Tunc-Ozcan, E. , Wert, S. L. , Lim, P. H. , Ferreira, A. , & Redei, E. E. (2018). Hippocampus-dependent memory and allele-specific gene expression in adult offspring of alcohol-consuming dams after neonatal treatment with thyroxin or metformin. Molecular Psychiatry, 23(7), 1643. 28727687 10.1038/mp.2017.129 PMC5775940

[brb31334-bib-0085] Van den Hove, D. , Kenis, G. , Brass, A. , Opstelten, R. , Rutten, B. , Bruschettini, M. , … Prickaerts, J. (2013). Vulnerability versus resilience to prenatal stress in male and female rats; implications from gene expression profiles in the hippocampus and frontal cortex. European Neuropsychopharmacology, 23(10), 1226–1246. 10.1016/j.euroneuro.2012.09.011 23199416

[brb31334-bib-0086] Wang, Z. , Gerstein, M. , & Snyder, M. (2009). RNA‐Seq: A revolutionary tool for transcriptomics. Nature Reviews Genetics, 10(1), 57 10.1038/nrg2484 PMC2949280 19015660

[brb31334-bib-0087] Willoughby, K. A. , Sheard, E. D. , Nash, K. , & Rovet, J. (2008). Effects of prenatal alcohol exposure on hippocampal volume, verbal learning, and verbal and spatial recall in late childhood. Journal of the International Neuropsychological Society, 14(6), 1022–1033. 10.1017/S1355617708081368 18954482

[brb31334-bib-0088] Wondrak, G. T. , Jacobson, M. K. , & Jacobson, E. L. (2005). Identification of quenchers of photoexcited states as novel agents for skin photoprotection. Journal of Pharmacology and Experimental Therapeutics, 312(2), 482–491. 10.1124/jpet.104.075101 15475591

